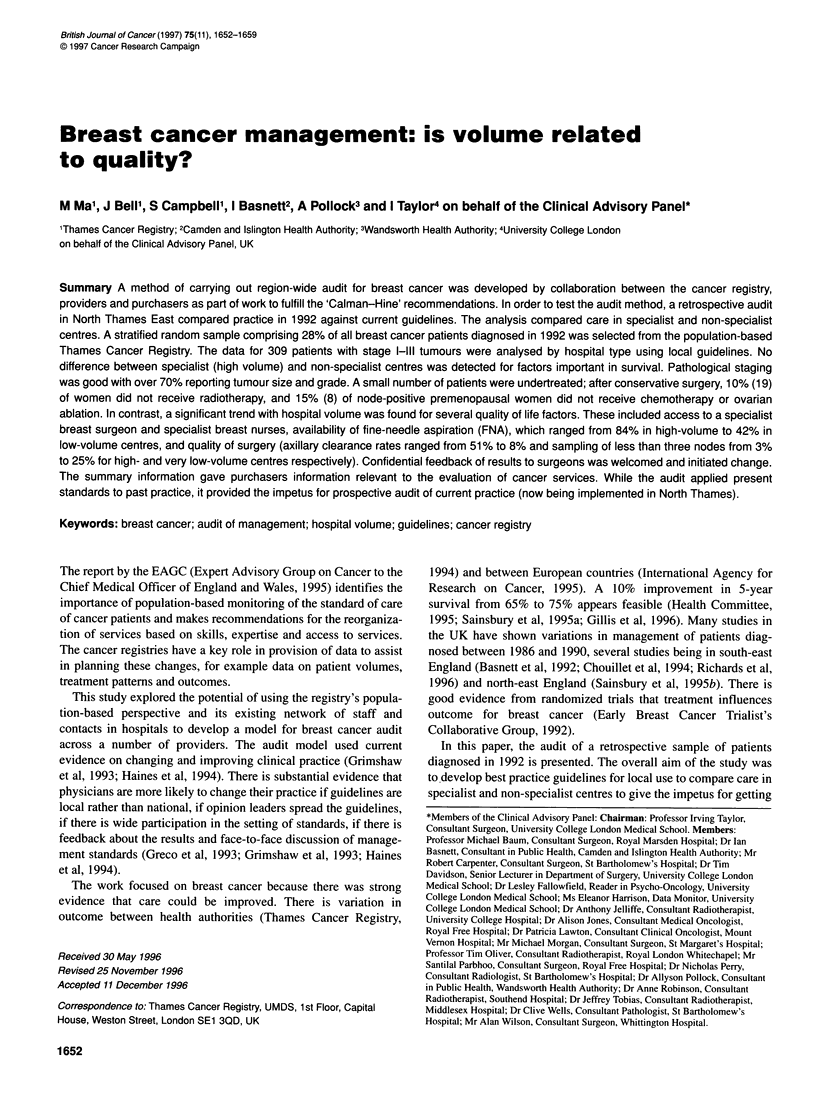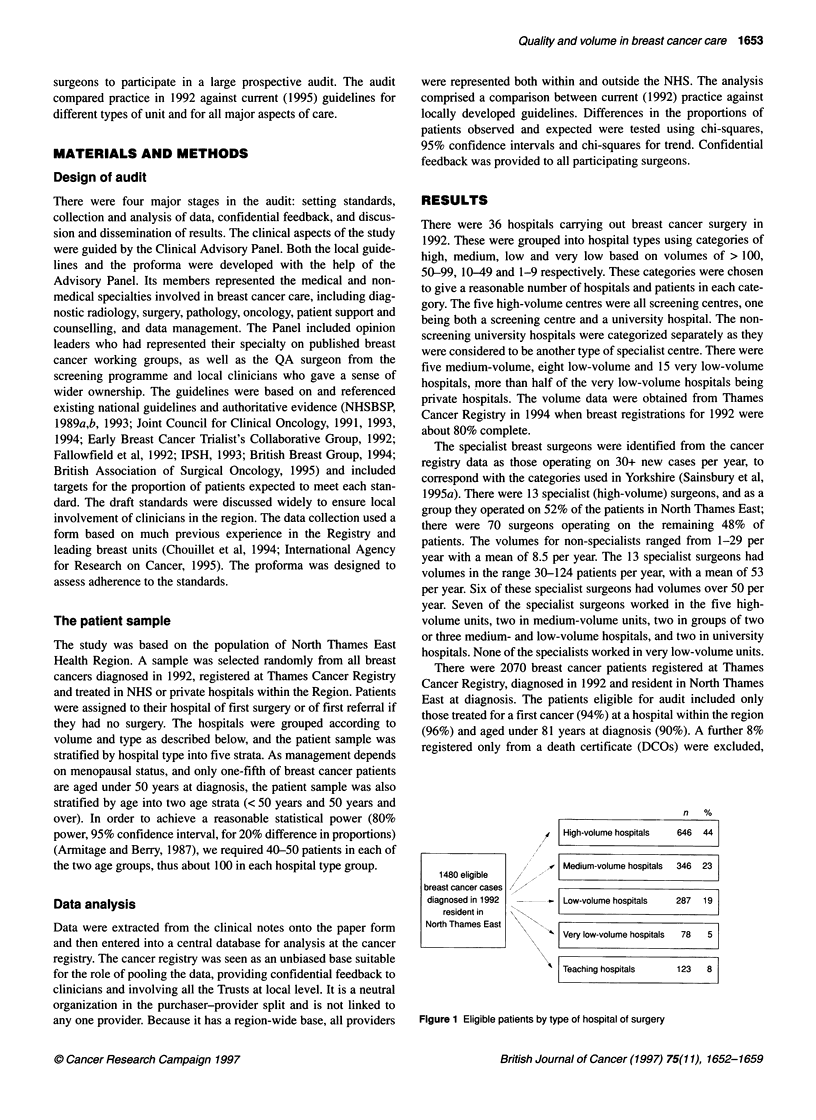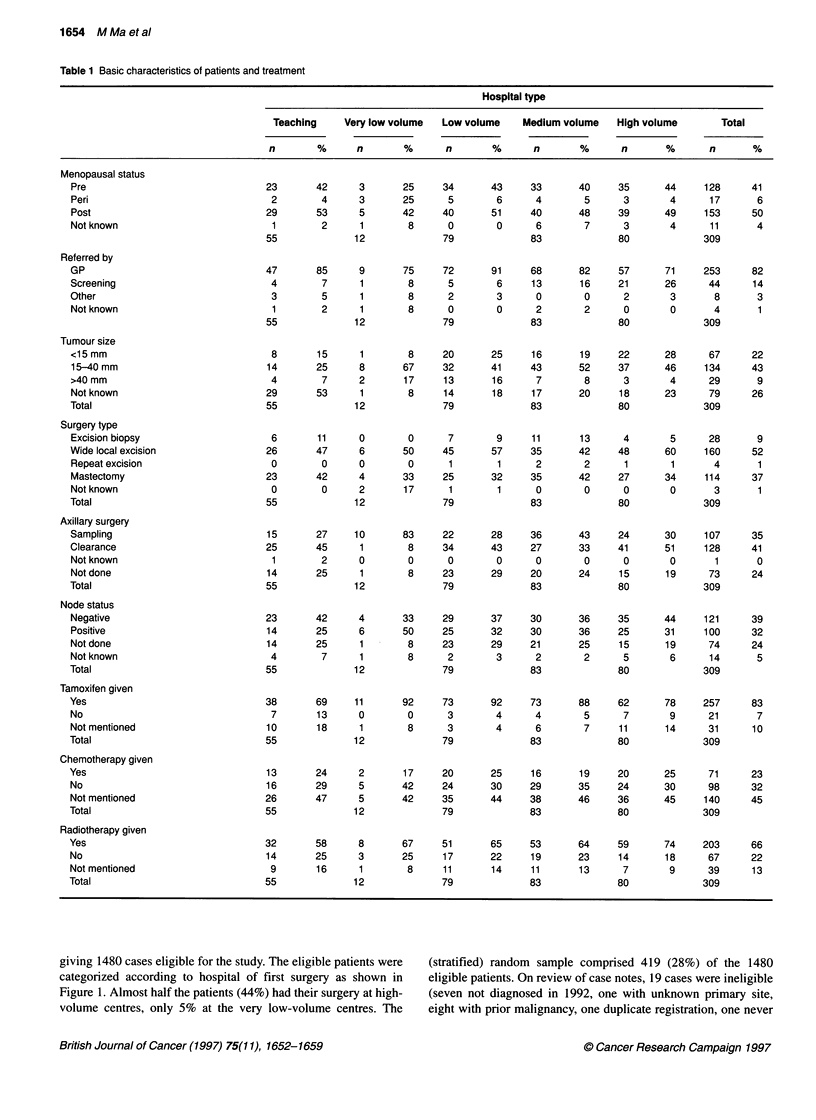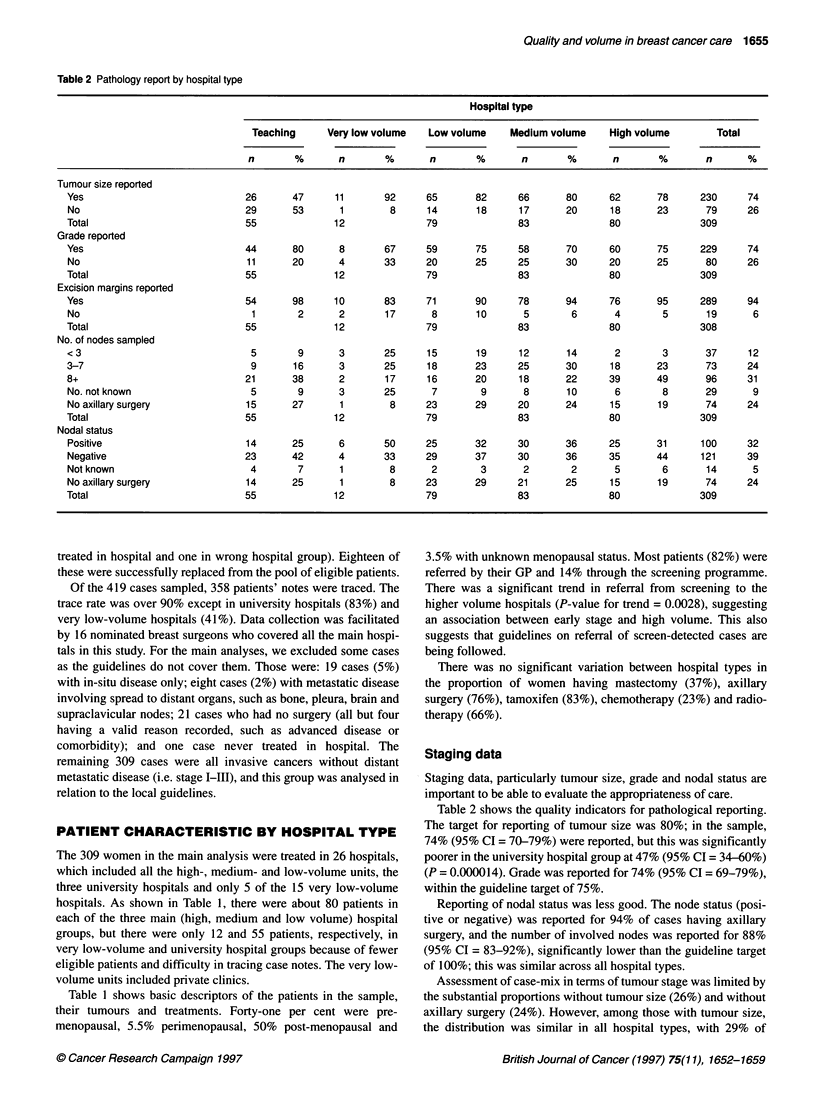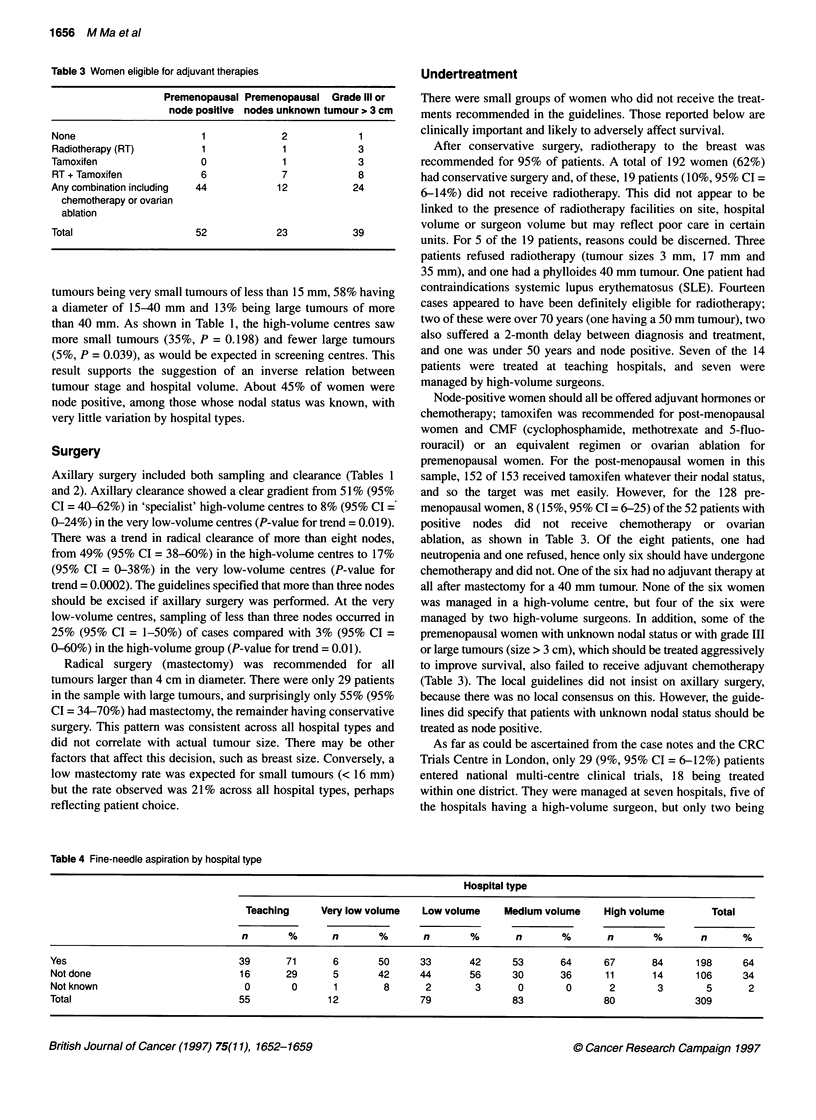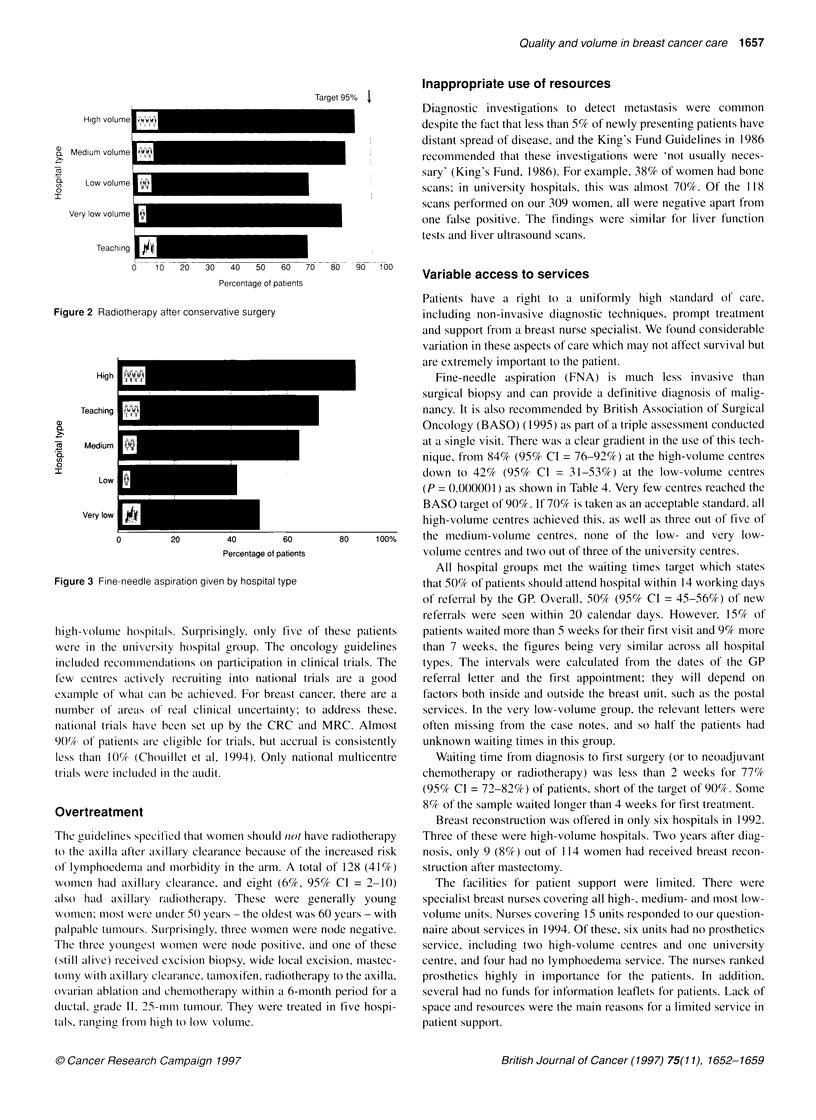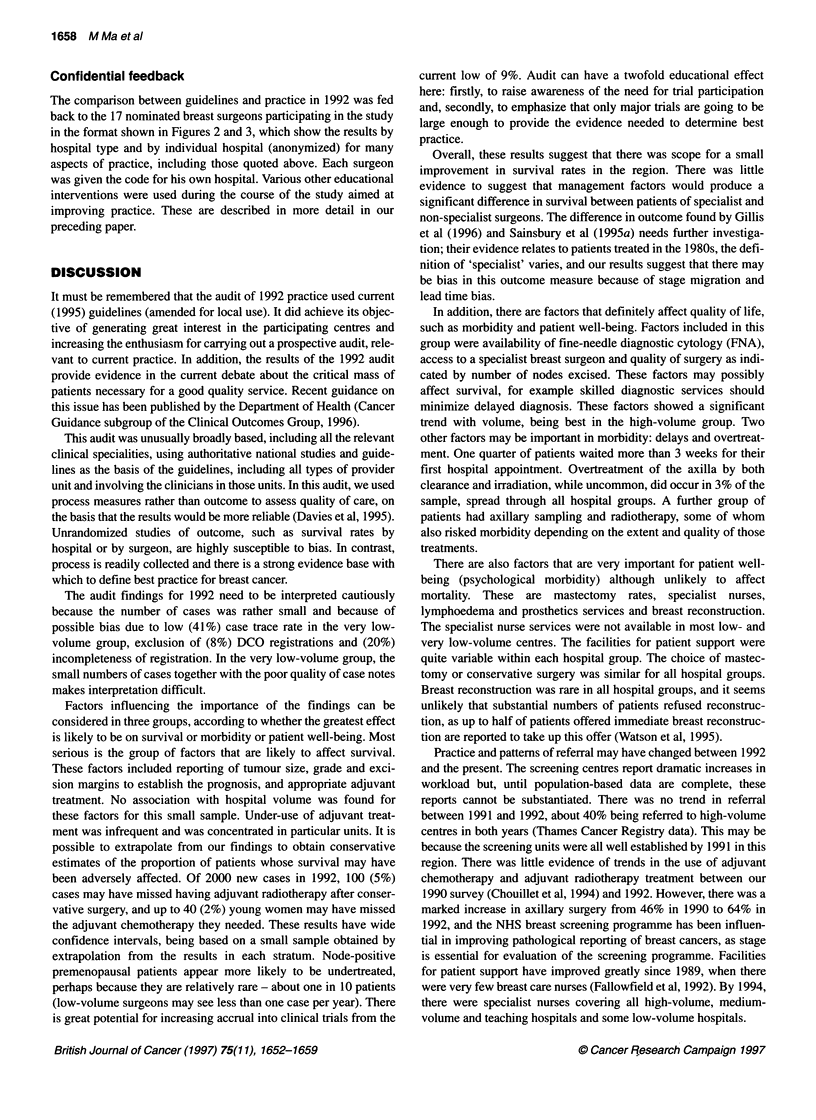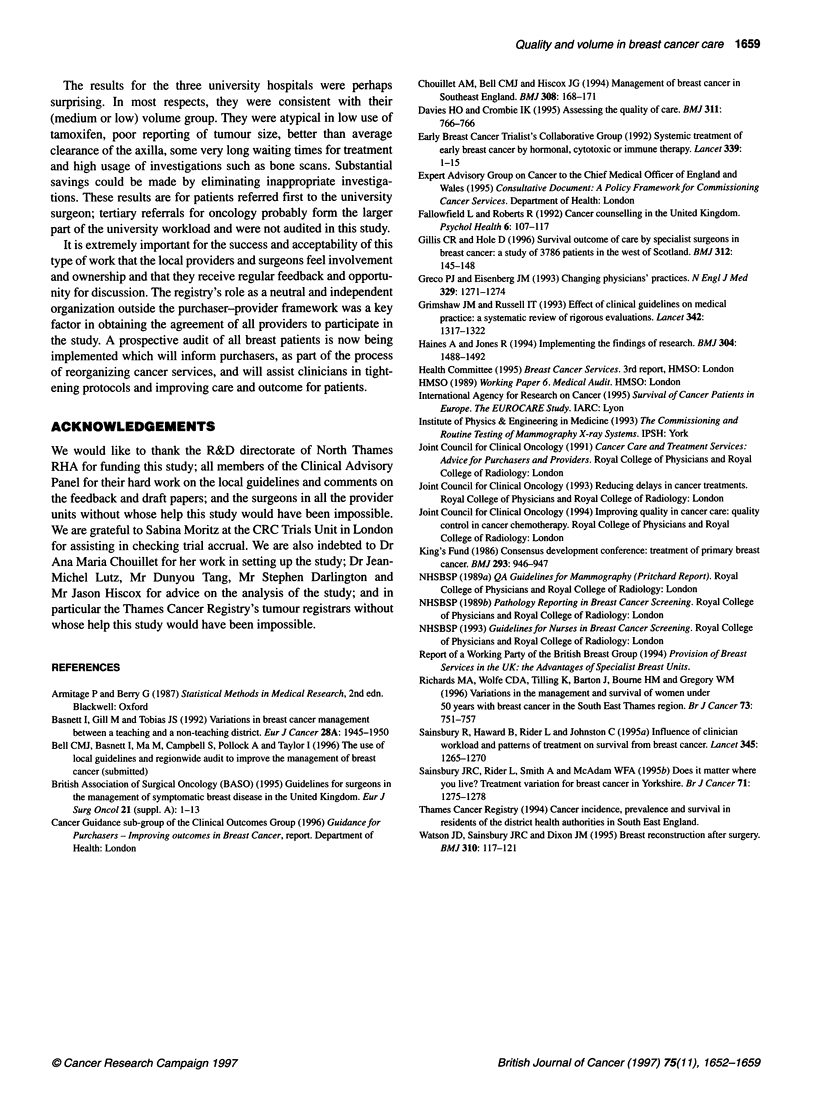# Breast cancer management: is volume related to quality? Clinical Advisory Panel.

**DOI:** 10.1038/bjc.1997.281

**Published:** 1997

**Authors:** M. Ma, J. Bell, S. Campbell, I. Basnett, A. Pollock, I. Taylor

**Affiliations:** Thames Cancer Registry, London, UK.

## Abstract

A method of carrying out region-wide audit for breast cancer was developed by collaboration between the cancer registry, providers and purchasers as part of work to fulfill the 'Calman-Hine' recommendations. In order to test the audit method, a retrospective audit in North Thames East compared practice in 1992 against current guidelines. The analysis compared care in specialist and non-specialist centres. A stratified random sample comprising 28% of all breast cancer patients diagnosed in 1992 was selected from the population-based Thames Cancer Registry. The data for 309 patients with stage I-III tumours were analysed by hospital type using local guidelines. No difference between specialist (high volume) and non-specialist centres was detected for factors important in survival. Pathological staging was good with over 70% reporting tumour size and grade. A small number of patients were undertreated; after conservative surgery, 10% (19) of women did not receive radiotherapy, and 15% (8) of node-positive premenopausal women did not receive chemotherapy or ovarian ablation. In contrast, a significant trend with hospital volume was found for several quality of life factors. These included access to a specialist breast surgeon and specialist breast nurses, availability of fine-needle aspiration (FNA), which ranged from 84% in high-volume to 42% in low-volume centres, and quality of surgery (axillary clearance rates ranged from 51% to 8% and sampling of less than three nodes from 3% to 25% for high- and very low-volume centres respectively). Confidential feedback of results to surgeons was welcomed and initiated change. The summary information gave purchasers information relevant to the evaluation of cancer services. While the audit applied present standards to past practice, it provided the impetus for prospective audit of current practice (now being implemented in North Thames).


British Journal of Cancer (1997) 75(11), 1652–1659
© 1997 Cancer Research Campaign

### Breast cancer management: is volume related to quality?

**M Ma[1], J Bell[1], S Campbell[1], I Basnett[2], A Pollock[3] and I Taylor[4] on behalf of the Clinical Advisory Panel***

[1]Thames Cancer Registry; [2]Camden and Islington Health Authority; [3]Wandsworth Health Authority; [4]University College London on behalf of the Clinical Advisory Panel, UK

**Summary** A method of carrying out region-wide audit for breast cancer was developed by collaboration between the cancer registry, providers and purchasers as part of work to fulfill the 'Calman–Hine' recommendations. In order to test the audit method, a retrospective audit in North Thames East compared practice in 1992 against current guidelines. The analysis compared care in specialist and non-specialist centres. A stratified random sample comprising 28% of all breast cancer patients diagnosed in 1992 was selected from the population-based Thames Cancer Registry. The data for 309 patients with stage I–III tumours were analysed by hospital type using local guidelines. No difference between specialist (high volume) and non-specialist centres was detected for factors important in survival. Pathological staging was good with over 70% reporting tumour size and grade. A small number of patients were undertreated; after conservative surgery, 10% (19) of women did not receive radiotherapy, and 15% (8) of node-positive premenopausal women did not receive chemotherapy or ovarian ablation. In contrast, a significant trend with hospital volume was found for several quality of life factors. These included access to a specialist breast surgeon and specialist breast nurses, availability of fine-needle aspiration (FNA), which ranged from 84% in high-volume to 42% in low-volume centres, and quality of surgery (axillary clearance rates ranged from 51% to 8% and sampling of less than three nodes from 3% to 25% for high- and very low-volume centres respectively). Confidential feedback of results to surgeons was welcomed and initiated change. The summary information gave purchasers information relevant to the evaluation of cancer services. While the audit applied present standards to past practice, it provided the impetus for prospective audit of current practice (now being implemented in North Thames).

**Keywords:** breast cancer; audit of management; hospital volume; guidelines; cancer registry

The report by the EAGC (Expert Advisory Group on Cancer to the Chief Medical Officer of England and Wales, 1995) identifies the importance of population-based monitoring of the standard of care of cancer patients and makes recommendations for the reorganization of services based on skills, expertise and access to services. The cancer registries have a key role in provision of data to assist in planning these changes, for example data on patient volumes, treatment patterns and outcomes.

This study explored the potential of using the registry's population-based perspective and its existing network of staff and contacts in hospitals to develop a model for breast cancer audit across a number of providers. The audit model used current evidence on changing and improving clinical practice (Grimshaw et al, 1993; Haines et al, 1994). There is substantial evidence that physicians are more likely to change their practice if guidelines are local rather than national, if opinion leaders spread the guidelines, if there is wide participation in the setting of standards, if there is feedback about the results and face-to-face discussion of management standards (Greco et al, 1993; Grimshaw et al, 1993; Haines et al, 1994).

The work focused on breast cancer because there was strong evidence that care could be improved. There is variation in outcome between health authorities (Thames Cancer Registry, 1994) and between European countries (International Agency for Research on Cancer, 1995). A 10% improvement in 5-year survival from 65% to 75% appears feasible (Health Committee, 1995; Sainsbury et al, 1995a; Gillis et al, 1996). Many studies in the UK have shown variations in management of patients diagnosed between 1986 and 1990, several studies being in south-east England (Basnett et al, 1992; Chouillet et al, 1994; Richards et al, 1996) and north-east England (Sainsbury et al, 1995*b*). There is good evidence from randomized trials that treatment influences outcome for breast cancer (Early Breast Cancer Trialist's Collaborative Group, 1992).

In this paper, the audit of a retrospective sample of patients diagnosed in 1992 is presented. The overall aim of the study was to develop best practice guidelines for local use to compare care in specialist and non-specialist centres to give the impetus for getting

*Received 30 May 1996*
*Revised 25 November 1996*
*Accepted 11 December 1996*

*Correspondence to:* Thames Cancer Registry, UMDS, 1st Floor, Capital House, Weston Street, London SE1 3QD, UK

*Members of the Clinical Advisory Panel: **Chairman**: Professor Irving Taylor, Consultant Surgeon, University College London Medical School. **Members**: Professor Michael Baum, Consultant Surgeon, Royal Marsden Hospital; Dr Ian Basnett, Consultant in Public Health, Camden and Islington Health Authority; Mr Robert Carpenter, Consultant Surgeon, St Bartholomew's Hospital; Dr Tim Davidson, Senior Lecturer in Department of Surgery, University College London Medical School; Dr Lesley Fallowfield, Reader in Psycho-Oncology, University College London Medical School; Ms Eleanor Harrison, Data Monitor, University College London Medical School; Dr Anthony Jelliffe, Consultant Radiotherapist, University College Hospital; Dr Alison Jones, Consultant Medical Oncologist, Royal Free Hospital; Dr Patricia Lawton, Consultant Clinical Oncologist, Mount Vernon Hospital; Mr Michael Morgan, Consultant Surgeon, St Margaret's Hospital; Professor Tim Oliver, Consultant Radiotherapist, Royal London Whitechapel; Mr Santilal Parbhoo, Consultant Surgeon, Royal Free Hospital; Dr Nicholas Perry, Consultant Radiologist, St Bartholomew's Hospital; Dr Allyson Pollock, Consultant in Public Health, Wandsworth Health Authority; Dr Anne Robinson, Consultant Radiotherapist, Southend Hospital; Dr Jeffrey Tobias, Consultant Radiotherapist, Middlesex Hospital; Dr Clive Wells, Consultant Pathologist, St Bartholomew's Hospital; Mr Alan Wilson, Consultant Surgeon, Whittington Hospital.

surgeons to participate in a large prospective audit. The audit compared practice in 1992 against current (1995) guidelines for different types of unit and for all major aspects of care.

### MATERIALS AND METHODS

### Design of audit

There were four major stages in the audit: setting standards, collection and analysis of data, confidential feedback, and discussion and dissemination of results. The clinical aspects of the study were guided by the Clinical Advisory Panel. Both the local guidelines and the proforma were developed with the help of the Advisory Panel. Its members represented the medical and non-medical specialties involved in breast cancer care, including diagnostic radiology, surgery, pathology, oncology, patient support and counselling, and data management. The Panel included opinion leaders who had represented their specialty on published breast cancer working groups, as well as the QA surgeon from the screening programme and local clinicians who gave a sense of wider ownership. The guidelines were based on and referenced existing national guidelines and authoritative evidence (NHSBSP, 1989*a*,*b*, 1993; Joint Council for Clinical Oncology, 1991, 1993, 1994; Early Breast Cancer Trialist's Collaborative Group, 1992; Fallowfield et al, 1992; IPSH, 1993; British Breast Group, 1994; British Association of Surgical Oncology, 1995) and included targets for the proportion of patients expected to meet each standard. The draft standards were discussed widely to ensure local involvement of clinicians in the region. The data collection used a form based on much previous experience in the Registry and leading breast units (Chouillet et al, 1994; International Agency for Research on Cancer, 1995). The proforma was designed to assess adherence to the standards.

### The patient sample

The study was based on the population of North Thames East Health Region. A sample was selected randomly from all breast cancers diagnosed in 1992, registered at Thames Cancer Registry and treated in NHS or private hospitals within the Region. Patients were assigned to their hospital of first surgery or of first referral if they had no surgery. The hospitals were grouped according to volume and type as described below, and the patient sample was stratified by hospital type into five strata. As management depends on menopausal status, and only one-fifth of breast cancer patients are aged under 50 years at diagnosis, the patient sample was also stratified by age into two age strata (< 50 years and 50 years and over). In order to achieve a reasonable statistical power (80% power, 95% confidence interval, for 20% difference in proportions) (Armitage and Berry, 1987), we required 40–50 patients in each of the two age groups, thus about 100 in each hospital type group.

### Data analysis

Data were extracted from the clinical notes onto the paper form and then entered into a central database for analysis at the cancer registry. The cancer registry was seen as an unbiased base suitable for the role of pooling the data, providing confidential feedback to clinicians and involving all the Trusts at local level. It is a neutral organization in the purchaser–provider split and is not linked to any one provider. Because it has a region-wide base, all providers were represented both within and outside the NHS. The analysis comprised a comparison between current (1992) practice against locally developed guidelines. Differences in the proportions of patients observed and expected were tested using chi-squares, 95% confidence intervals and chi-squares for trend. Confidential feedback was provided to all participating surgeons.

### RESULTS

There were 36 hospitals carrying out breast cancer surgery in 1992. These were grouped into hospital types using categories of high, medium, low and very low based on volumes of > 100, 50–99, 10–49 and 1–9 respectively. These categories were chosen to give a reasonable number of hospitals and patients in each category. The five high-volume centres were all screening centres, one being both a screening centre and a university hospital. The non-screening university hospitals were categorized separately as they were considered to be another type of specialist centre. There were five medium-volume, eight low-volume and 15 very low-volume hospitals, more than half of the very low-volume hospitals being private hospitals. The volume data were obtained from Thames Cancer Registry in 1994 when breast registrations for 1992 were about 80% complete.

The specialist breast surgeons were identified from the cancer registry data as those operating on 30+ new cases per year, to correspond with the categories used in Yorkshire (Sainsbury et al, 1995*a*). There were 13 specialist (high-volume) surgeons, and as a group they operated on 52% of the patients in North Thames East; there were 70 surgeons operating on the remaining 48% of patients. The volumes for non-specialists ranged from 1–29 per year with a mean of 8.5 per year. The 13 specialist surgeons had volumes in the range 30–124 patients per year, with a mean of 53 per year. Six of these specialist surgeons had volumes over 50 per year. Seven of the specialist surgeons worked in the five high-volume units, two in medium-volume units, two in groups of two or three medium- and low-volume hospitals, and two in university hospitals. None of the specialists worked in very low-volume units.

There were 2070 breast cancer patients registered at Thames Cancer Registry, diagnosed in 1992 and resident in North Thames East at diagnosis. The patients eligible for audit included only those treated for a first cancer (94%) at a hospital within the region (96%) and aged under 81 years at diagnosis (90%). A further 8% registered only from a death certificate (DCOs) were excluded,

**Figure 1** Eligible patients by type of hospital of surgery

© Cancer Research Campaign 1997

**Table 1** Basic characteristics of patients and treatment

| | Hospital type | | | | | | | | | | | |
|---|---|---|---|---|---|---|---|---|---|---|---|---|
| | Teaching | | Very low volume | | Low volume | | Medium volume | | High volume | | Total | |
| | *n* | % | *n* | % | *n* | % | *n* | % | *n* | % | *n* | % |
| Menopausal status | | | | | | | | | | | | |
| Pre | 23 | 42 | 3 | 25 | 34 | 43 | 33 | 40 | 35 | 44 | 128 | 41 |
| Peri | 2 | 4 | 3 | 25 | 5 | 6 | 4 | 5 | 3 | 4 | 17 | 6 |
| Post | 29 | 53 | 5 | 42 | 40 | 51 | 40 | 48 | 39 | 49 | 153 | 50 |
| Not known | 1 | 2 | 1 | 8 | 0 | 0 | 6 | 7 | 3 | 4 | 11 | 4 |
| | 55 | | 12 | | 79 | | 83 | | 80 | | 309 | |
| Referred by | | | | | | | | | | | | |
| GP | 47 | 85 | 9 | 75 | 72 | 91 | 68 | 82 | 57 | 71 | 253 | 82 |
| Screening | 4 | 7 | 1 | 8 | 5 | 6 | 13 | 16 | 21 | 26 | 44 | 14 |
| Other | 3 | 5 | 1 | 8 | 2 | 3 | 0 | 0 | 2 | 3 | 8 | 3 |
| Not known | 1 | 2 | 1 | 8 | 0 | 0 | 2 | 2 | 0 | 0 | 4 | 1 |
| | 55 | | 12 | | 79 | | 83 | | 80 | | 309 | |
| Tumour size | | | | | | | | | | | | |
| <15 mm | 8 | 15 | 1 | 8 | 20 | 25 | 16 | 19 | 22 | 28 | 67 | 22 |
| 15–40 mm | 14 | 25 | 8 | 67 | 32 | 41 | 43 | 52 | 37 | 46 | 134 | 43 |
| >40 mm | 4 | 7 | 2 | 17 | 13 | 16 | 7 | 8 | 3 | 4 | 29 | 9 |
| Not known | 29 | 53 | 1 | 8 | 14 | 18 | 17 | 20 | 18 | 23 | 79 | 26 |
| Total | 55 | | 12 | | 79 | | 83 | | 80 | | 309 | |
| Surgery type | | | | | | | | | | | | |
| Excision biopsy | 6 | 11 | 0 | 0 | 7 | 9 | 11 | 13 | 4 | 5 | 28 | 9 |
| Wide local excision | 26 | 47 | 6 | 50 | 45 | 57 | 35 | 42 | 48 | 60 | 160 | 52 |
| Repeat excision | 0 | 0 | 0 | 0 | 1 | 1 | 2 | 2 | 1 | 1 | 4 | 1 |
| Mastectomy | 23 | 42 | 4 | 33 | 25 | 32 | 35 | 42 | 27 | 34 | 114 | 37 |
| Not known | 0 | 0 | 2 | 17 | 1 | 1 | 0 | 0 | 0 | 0 | 3 | 1 |
| Total | 55 | | 12 | | 79 | | 83 | | 80 | | 309 | |
| Axillary surgery | | | | | | | | | | | | |
| Sampling | 15 | 27 | 10 | 83 | 22 | 28 | 36 | 43 | 24 | 30 | 107 | 35 |
| Clearance | 25 | 45 | 1 | 8 | 34 | 43 | 27 | 33 | 41 | 51 | 128 | 41 |
| Not known | 1 | 2 | 0 | 0 | 0 | 0 | 0 | 0 | 0 | 0 | 1 | 0 |
| Not done | 14 | 25 | 1 | 8 | 23 | 29 | 20 | 24 | 15 | 19 | 73 | 24 |
| Total | 55 | | 12 | | 79 | | 83 | | 80 | | 309 | |
| Node status | | | | | | | | | | | | |
| Negative | 23 | 42 | 4 | 33 | 29 | 37 | 30 | 36 | 35 | 44 | 121 | 39 |
| Positive | 14 | 25 | 6 | 50 | 25 | 32 | 30 | 36 | 25 | 31 | 100 | 32 |
| Not done | 14 | 25 | 1 | 8 | 23 | 29 | 21 | 25 | 15 | 19 | 74 | 24 |
| Not known | 4 | 7 | 1 | 8 | 2 | 3 | 2 | 2 | 5 | 6 | 14 | 5 |
| Total | 55 | | 12 | | 79 | | 83 | | 80 | | 309 | |
| Tamoxifen given | | | | | | | | | | | | |
| Yes | 38 | 69 | 11 | 92 | 73 | 92 | 73 | 88 | 62 | 78 | 257 | 83 |
| No | 7 | 13 | 0 | 0 | 3 | 4 | 4 | 5 | 7 | 9 | 21 | 7 |
| Not mentioned | 10 | 18 | 1 | 8 | 3 | 4 | 6 | 7 | 11 | 14 | 31 | 10 |
| Total | 55 | | 12 | | 79 | | 83 | | 80 | | 309 | |
| Chemotherapy given | | | | | | | | | | | | |
| Yes | 13 | 24 | 2 | 17 | 20 | 25 | 16 | 19 | 20 | 25 | 71 | 23 |
| No | 16 | 29 | 5 | 42 | 24 | 30 | 29 | 35 | 24 | 30 | 98 | 32 |
| Not mentioned | 26 | 47 | 5 | 42 | 35 | 44 | 38 | 46 | 36 | 45 | 140 | 45 |
| Total | 55 | | 12 | | 79 | | 83 | | 80 | | 309 | |
| Radiotherapy given | | | | | | | | | | | | |
| Yes | 32 | 58 | 8 | 67 | 51 | 65 | 53 | 64 | 59 | 74 | 203 | 66 |
| No | 14 | 25 | 3 | 25 | 17 | 22 | 19 | 23 | 14 | 18 | 67 | 22 |
| Not mentioned | 9 | 16 | 1 | 8 | 11 | 14 | 11 | 13 | 7 | 9 | 39 | 13 |
| Total | 55 | | 12 | | 79 | | 83 | | 80 | | 309 | |

giving 1480 cases eligible for the study. The eligible patients were categorized according to hospital of first surgery as shown in Figure 1. Almost half the patients (44%) had their surgery at high-volume centres, only 5% at the very low-volume centres. The (stratified) random sample comprised 419 (28%) of the 1480 eligible patients. On review of case notes, 19 cases were ineligible (seven not diagnosed in 1992, one with unknown primary site, eight with prior malignancy, one duplicate registration, one never

*© Cancer Research Campaign 1997*

**Table 2** Pathology report by hospital type

| | Hospital type | | | | | | | | | | | |
|---|---|---|---|---|---|---|---|---|---|---|---|---|
| | Teaching | | Very low volume | | Low volume | | Medium volume | | High volume | | Total | |
| | *n* | % | *n* | % | *n* | % | *n* | % | *n* | % | *n* | % |
| Tumour size reported | | | | | | | | | | | | |
| Yes | 26 | 47 | 11 | 92 | 65 | 82 | 66 | 80 | 62 | 78 | 230 | 74 |
| No | 29 | 53 | 1 | 8 | 14 | 18 | 17 | 20 | 18 | 23 | 79 | 26 |
| Total | 55 | | 12 | | 79 | | 83 | | 80 | | 309 | |
| Grade reported | | | | | | | | | | | | |
| Yes | 44 | 80 | 8 | 67 | 59 | 75 | 58 | 70 | 60 | 75 | 229 | 74 |
| No | 11 | 20 | 4 | 33 | 20 | 25 | 25 | 30 | 20 | 25 | 80 | 26 |
| Total | 55 | | 12 | | 79 | | 83 | | 80 | | 309 | |
| Excision margins reported | | | | | | | | | | | | |
| Yes | 54 | 98 | 10 | 83 | 71 | 90 | 78 | 94 | 76 | 95 | 289 | 94 |
| No | 1 | 2 | 2 | 17 | 8 | 10 | 5 | 6 | 4 | 5 | 19 | 6 |
| Total | 55 | | 12 | | 79 | | 83 | | 80 | | 308 | |
| No. of nodes sampled | | | | | | | | | | | | |
| < 3 | 5 | 9 | 3 | 25 | 15 | 19 | 12 | 14 | 2 | 3 | 37 | 12 |
| 3–7 | 9 | 16 | 3 | 25 | 18 | 23 | 25 | 30 | 18 | 23 | 73 | 24 |
| 8+ | 21 | 38 | 2 | 17 | 16 | 20 | 18 | 22 | 39 | 49 | 96 | 31 |
| No. not known | 5 | 9 | 3 | 25 | 7 | 9 | 8 | 10 | 6 | 8 | 29 | 9 |
| No axillary surgery | 15 | 27 | 1 | 8 | 23 | 29 | 20 | 24 | 15 | 19 | 74 | 24 |
| Total | 55 | | 12 | | 79 | | 83 | | 80 | | 309 | |
| Nodal status | | | | | | | | | | | | |
| Positive | 14 | 25 | 6 | 50 | 25 | 32 | 30 | 36 | 25 | 31 | 100 | 32 |
| Negative | 23 | 42 | 4 | 33 | 29 | 37 | 30 | 36 | 35 | 44 | 121 | 39 |
| Not known | 4 | 7 | 1 | 8 | 2 | 3 | 2 | 2 | 5 | 6 | 14 | 5 |
| No axillary surgery | 14 | 25 | 1 | 8 | 23 | 29 | 21 | 25 | 15 | 19 | 74 | 24 |
| Total | 55 | | 12 | | 79 | | 83 | | 80 | | 309 | |

treated in hospital and one in wrong hospital group). Eighteen of these were successfully replaced from the pool of eligible patients.

Of the 419 cases sampled, 358 patients' notes were traced. The trace rate was over 90% except in university hospitals (83%) and very low-volume hospitals (41%). Data collection was facilitated by 16 nominated breast surgeons who covered all the main hospitals in this study. For the main analyses, we excluded some cases as the guidelines do not cover them. Those were: 19 cases (5%) with in-situ disease only; eight cases (2%) with metastatic disease involving spread to distant organs, such as bone, pleura, brain and supraclavicular nodes; 21 cases who had no surgery (all but four having a valid reason recorded, such as advanced disease or comorbidity); and one case never treated in hospital. The remaining 309 cases were all invasive cancers without distant metastatic disease (i.e. stage I–III), and this group was analysed in relation to the local guidelines.

### PATIENT CHARACTERISTIC BY HOSPITAL TYPE

The 309 women in the main analysis were treated in 26 hospitals, which included all the high-, medium- and low-volume units, the three university hospitals and only 5 of the 15 very low-volume hospitals. As shown in Table 1, there were about 80 patients in each of the three main (high, medium and low volume) hospital groups, but there were only 12 and 55 patients, respectively, in very low-volume and university hospital groups because of fewer eligible patients and difficulty in tracing case notes. The very low-volume units included private clinics.

Table 1 shows basic descriptors of the patients in the sample, their tumours and treatments. Forty-one per cent were pre-menopausal, 5.5% perimenopausal, 50% post-menopausal and 3.5% with unknown menopausal status. Most patients (82%) were referred by their GP and 14% through the screening programme. There was a significant trend in referral from screening to the higher volume hospitals (*P*-value for trend = 0.0028), suggesting an association between early stage and high volume. This also suggests that guidelines on referral of screen-detected cases are being followed.

There was no significant variation between hospital types in the proportion of women having mastectomy (37%), axillary surgery (76%), tamoxifen (83%), chemotherapy (23%) and radiotherapy (66%).

### Staging data

Staging data, particularly tumour size, grade and nodal status are important to be able to evaluate the appropriateness of care.

Table 2 shows the quality indicators for pathological reporting. The target for reporting of tumour size was 80%; in the sample, 74% (95% CI = 70–79%) were reported, but this was significantly poorer in the university hospital group at 47% (95% CI = 34–60%) ($P = 0.000014$). Grade was reported for 74% (95% CI = 69–79%), within the guideline target of 75%.

Reporting of nodal status was less good. The node status (positive or negative) was reported for 94% of cases having axillary surgery, and the number of involved nodes was reported for 88% (95% CI = 83–92%), significantly lower than the guideline target of 100%; this was similar across all hospital types.

Assessment of case-mix in terms of tumour stage was limited by the substantial proportions without tumour size (26%) and without axillary surgery (24%). However, among those with tumour size, the distribution was similar in all hospital types, with 29% of

© Cancer Research Campaign 1997

Table 3 Women eligible for adjuvant therapies

| | Premenopausal node positive | Premenopausal nodes unknown | Grade III or tumour > 3 cm |
|---|---|---|---|
| None | 1 | 2 | 1 |
| Radiotherapy (RT) | 1 | 1 | 3 |
| Tamoxifen | 0 | 1 | 3 |
| RT + Tamoxifen | 6 | 7 | 8 |
| Any combination including chemotherapy or ovarian ablation | 44 | 12 | 24 |
| Total | 52 | 23 | 39 |

tumours being very small tumours of less than 15 mm, 58% having a diameter of 15–40 mm and 13% being large tumours of more than 40 mm. As shown in Table 1, the high-volume centres saw more small tumours (35%, $P$ = 0.198) and fewer large tumours (5%, $P$ = 0.039), as would be expected in screening centres. This result supports the suggestion of an inverse relation between tumour stage and hospital volume. About 45% of women were node positive, among those whose nodal status was known, with very little variation by hospital types.

### Surgery

Axillary surgery included both sampling and clearance (Tables 1 and 2). Axillary clearance showed a clear gradient from 51% (95% CI = 40–62%) in 'specialist' high-volume centres to 8% (95% CI = 0–24%) in the very low-volume centres ($P$-value for trend = 0.019). There was a trend in radical clearance of more than eight nodes, from 49% (95% CI = 38–60%) in the high-volume centres to 17% (95% CI = 0–38%) in the very low-volume centres ($P$-value for trend = 0.0002). The guidelines specified that more than three nodes should be excised if axillary surgery was performed. At the very low-volume centres, sampling of less than three nodes occurred in 25% (95% CI = 1–50%) of cases compared with 3% (95% CI = 0–60%) in the high-volume group ($P$-value for trend = 0.01).

Radical surgery (mastectomy) was recommended for all tumours larger than 4 cm in diameter. There were only 29 patients in the sample with large tumours, and surprisingly only 55% (95% CI = 34–70%) had mastectomy, the remainder having conservative surgery. This pattern was consistent across all hospital types and did not correlate with actual tumour size. There may be other factors that affect this decision, such as breast size. Conversely, a low mastectomy rate was expected for small tumours (< 16 mm) but the rate observed was 21% across all hospital types, perhaps reflecting patient choice.

### Undertreatment

There were small groups of women who did not receive the treatments recommended in the guidelines. Those reported below are clinically important and likely to adversely affect survival.

After conservative surgery, radiotherapy to the breast was recommended for 95% of patients. A total of 192 women (62%) had conservative surgery and, of these, 19 patients (10%, 95% CI = 6–14%) did not receive radiotherapy. This did not appear to be linked to the presence of radiotherapy facilities on site, hospital volume or surgeon volume but may reflect poor care in certain units. For 5 of the 19 patients, reasons could be discerned. Three patients refused radiotherapy (tumour sizes 3 mm, 17 mm and 35 mm), and one had a phylloides 40 mm tumour. One patient had contraindications systemic lupus erythematosus (SLE). Fourteen cases appeared to have been definitely eligible for radiotherapy; two of these were over 70 years (one having a 50 mm tumour), two also suffered a 2-month delay between diagnosis and treatment, and one was under 50 years and node positive. Seven of the 14 patients were treated at teaching hospitals, and seven were managed by high-volume surgeons.

Node-positive women should all be offered adjuvant hormones or chemotherapy; tamoxifen was recommended for post-menopausal women and CMF (cyclophosphamide, methotrexate and 5-fluorouracil) or an equivalent regimen or ovarian ablation for premenopausal women. For the post-menopausal women in this sample, 152 of 153 received tamoxifen whatever their nodal status, and so the target was met easily. However, for the 128 premenopausal women, 8 (15%, 95% CI = 6–25) of the 52 patients with positive nodes did not receive chemotherapy or ovarian ablation, as shown in Table 3. Of the eight patients, one had neutropenia and one refused, hence only six should have undergone chemotherapy and did not. One of the six had no adjuvant therapy at all after mastectomy for a 40 mm tumour. None of the six women was managed in a high-volume centre, but four of the six were managed by two high-volume surgeons. In addition, some of the premenopausal women with unknown nodal status or with grade III or large tumours (size > 3 cm), which should be treated aggressively to improve survival, also failed to receive adjuvant chemotherapy (Table 3). The local guidelines did not insist on axillary surgery, because there was no local consensus on this. However, the guidelines did specify that patients with unknown nodal status should be treated as node positive.

As far as could be ascertained from the case notes and the CRC Trials Centre in London, only 29 (9%, 95% CI = 6–12%) patients entered national multi-centre clinical trials, 18 being treated within one district. They were managed at seven hospitals, five of the hospitals having a high-volume surgeon, but only two being

Table 4 Fine-needle aspiration by hospital type

| | Hospital type | | | | | | | | | | | |
|---|---|---|---|---|---|---|---|---|---|---|---|---|
| | Teaching | | Very low volume | | Low volume | | Medium volume | | High volume | | Total | |
| | *n* | % | *n* | % | *n* | % | *n* | % | *n* | % | *n* | % |
| Yes | 39 | 71 | 6 | 50 | 33 | 42 | 53 | 64 | 67 | 84 | 198 | 64 |
| Not done | 16 | 29 | 5 | 42 | 44 | 56 | 30 | 36 | 11 | 14 | 106 | 34 |
| Not known | 0 | 0 | 1 | 8 | 2 | 3 | 0 | 0 | 2 | 3 | 5 | 2 |
| Total | 55 | | 12 | | 79 | | 83 | | 80 | | 309 | |

© Cancer Research Campaign 1997

**Figure 2** Radiotherapy after conservative surgery

**Figure 3** Fine-needle aspiration given by hospital type high-volume hospitals. Surprisingly, only five of these patients were in the university hospital group. The oncology guidelines included recommendations on participation in clinical trials. The few centres actively recruiting into national trials are a good example of what can be achieved. For breast cancer, there are a number of areas of real clinical uncertainty; to address these, national trials have been set up by the CRC and MRC. Almost 90% of patients are eligible for trials, but accrual is consistently less than 10% (Chouillet et al, 1994). Only national multicentre trials were included in the audit.

### Overtreatment

The guidelines specified that women should *not* have radiotherapy to the axilla after axillary clearance because of the increased risk of lymphoedema and morbidity in the arm. A total of 128 (41%) women had axillary clearance, and eight (6%, 95% CI = 2–10) also had axillary radiotherapy. These were generally young women; most were under 50 years – the oldest was 60 years – with palpable tumours. Surprisingly, three women were node negative. The three youngest women were node positive, and one of these (still alive) received excision biopsy, wide local excision, mastectomy with axillary clearance, tamoxifen, radiotherapy to the axilla, ovarian ablation and chemotherapy within a 6-month period for a ductal, grade II, 25-mm tumour. They were treated in five hospitals, ranging from high to low volume.

### Inappropriate use of resources

Diagnostic investigations to detect metastasis were common despite the fact that less than 5% of newly presenting patients have distant spread of disease, and the King's Fund Guidelines in 1986 recommended that these investigations were 'not usually necessary' (King's Fund, 1986). For example, 38% of women had bone scans; in university hospitals, this was almost 70%. Of the 118 scans performed on our 309 women, all were negative apart from one false positive. The findings were similar for liver function tests and liver ultrasound scans.

### Variable access to services

Patients have a right to a uniformly high standard of care, including non-invasive diagnostic techniques, prompt treatment and support from a breast nurse specialist. We found considerable variation in these aspects of care which may not affect survival but are extremely important to the patient.

Fine-needle aspiration (FNA) is much less invasive than surgical biopsy and can provide a definitive diagnosis of malignancy. It is also recommended by British Association of Surgical Oncology (BASO) (1995) as part of a triple assessment conducted at a single visit. There was a clear gradient in the use of this technique, from 84% (95% CI = 76–92%) at the high-volume centres down to 42% (95% CI = 31–53%) at the low-volume centres ($P = 0.000001$) as shown in Table 4. Very few centres reached the BASO target of 90%. If 70% is taken as an acceptable standard, all high-volume centres achieved this, as well as three out of five of the medium-volume centres, none of the low- and very low-volume centres and two out of three of the university centres.

All hospital groups met the waiting times target which states that 50% of patients should attend hospital within 14 working days of referral by the GP. Overall, 50% (95% CI = 45–56%) of new referrals were seen within 20 calendar days. However, 15% of patients waited more than 5 weeks for their first visit and 9% more than 7 weeks, the figures being very similar across all hospital types. The intervals were calculated from the dates of the GP referral letter and the first appointment; they will depend on factors both inside and outside the breast unit, such as the postal services. In the very low-volume group, the relevant letters were often missing from the case notes, and so half the patients had unknown waiting times in this group.

Waiting time from diagnosis to first surgery (or to neoadjuvant chemotherapy or radiotherapy) was less than 2 weeks for 77% (95% CI = 72–82%) of patients, short of the target of 90%. Some 8% of the sample waited longer than 4 weeks for first treatment.

Breast reconstruction was offered in only six hospitals in 1992. Three of these were high-volume hospitals. Two years after diagnosis, only 9 (8%) out of 114 women had received breast reconstruction after mastectomy.

The facilities for patient support were limited. There were specialist breast nurses covering all high-, medium- and most low-volume units. Nurses covering 15 units responded to our questionnaire about services in 1994. Of these, six units had no prosthetics service, including two high-volume centres and one university centre, and four had no lymphoedema service. The nurses ranked prosthetics highly in importance for the patients. In addition, several had no funds for information leaflets for patients. Lack of space and resources were the main reasons for a limited service in patient support.

© Cancer Research Campaign 1997

### Confidential feedback

The comparison between guidelines and practice in 1992 was fed back to the 17 nominated breast surgeons participating in the study in the format shown in Figures 2 and 3, which show the results by hospital type and by individual hospital (anonymized) for many aspects of practice, including those quoted above. Each surgeon was given the code for his own hospital. Various other educational interventions were used during the course of the study aimed at improving practice. These are described in more detail in our preceding paper.

### DISCUSSION

It must be remembered that the audit of 1992 practice used current (1995) guidelines (amended for local use). It did achieve its objective of generating great interest in the participating centres and increasing the enthusiasm for carrying out a prospective audit, relevant to current practice. In addition, the results of the 1992 audit provide evidence in the current debate about the critical mass of patients necessary for a good quality service. Recent guidance on this issue has been published by the Department of Health (Cancer Guidance subgroup of the Clinical Outcomes Group, 1996).

This audit was unusually broadly based, including all the relevant clinical specialities, using authoritative national studies and guidelines as the basis of the guidelines, including all types of provider unit and involving the clinicians in those units. In this audit, we used process measures rather than outcome to assess quality of care, on the basis that the results would be more reliable (Davies et al, 1995). Unrandomized studies of outcome, such as survival rates by hospital or by surgeon, are highly susceptible to bias. In contrast, process is readily collected and there is a strong evidence base with which to define best practice for breast cancer.

The audit findings for 1992 need to be interpreted cautiously because the number of cases was rather small and because of possible bias due to low (41%) case trace rate in the very low-volume group, exclusion of (8%) DCO registrations and (20%) incompleteness of registration. In the very low-volume group, the small numbers of cases together with the poor quality of case notes makes interpretation difficult.

Factors influencing the importance of the findings can be considered in three groups, according to whether the greatest effect is likely to be on survival or morbidity or patient well-being. Most serious is the group of factors that are likely to affect survival. These factors included reporting of tumour size, grade and excision margins to establish the prognosis, and appropriate adjuvant treatment. No association with hospital volume was found for these factors for this small sample. Under-use of adjuvant treatment was infrequent and was concentrated in particular units. It is possible to extrapolate from our findings to obtain conservative estimates of the proportion of patients whose survival may have been adversely affected. Of 2000 new cases in 1992, 100 (5%) cases may have missed having adjuvant radiotherapy after conservative surgery, and up to 40 (2%) young women may have missed the adjuvant chemotherapy they needed. These results have wide confidence intervals, being based on a small sample obtained by extrapolation from the results in each stratum. Node-positive premenopausal patients appear more likely to be undertreated, perhaps because they are relatively rare – about one in 10 patients (low-volume surgeons may see less than one case per year). There is great potential for increasing accrual into clinical trials from the current low of 9%. Audit can have a twofold educational effect here: firstly, to raise awareness of the need for trial participation and, secondly, to emphasize that only major trials are going to be large enough to provide the evidence needed to determine best practice.

Overall, these results suggest that there was scope for a small improvement in survival rates in the region. There was little evidence to suggest that management factors would produce a significant difference in survival between patients of specialist and non-specialist surgeons. The difference in outcome found by Gillis et al (1996) and Sainsbury et al (1995*a*) needs further investigation; their evidence relates to patients treated in the 1980s, the definition of 'specialist' varies, and our results suggest that there may be bias in this outcome measure because of stage migration and lead time bias.

In addition, there are factors that definitely affect quality of life, such as morbidity and patient well-being. Factors included in this group were availability of fine-needle diagnostic cytology (FNA), access to a specialist breast surgeon and quality of surgery as indicated by number of nodes excised. These factors may possibly affect survival, for example skilled diagnostic services should minimize delayed diagnosis. These factors showed a significant trend with volume, being best in the high-volume group. Two other factors may be important in morbidity: delays and overtreatment. One quarter of patients waited more than 3 weeks for their first hospital appointment. Overtreatment of the axilla by both clearance and irradiation, while uncommon, did occur in 3% of the sample, spread through all hospital groups. A further group of patients had axillary sampling and radiotherapy, some of whom also risked morbidity depending on the extent and quality of those treatments.

There are also factors that are very important for patient well-being (psychological morbidity) although unlikely to affect mortality. These are mastectomy rates, specialist nurses, lymphoedema and prosthetics services and breast reconstruction. The specialist nurse services were not available in most low- and very low-volume centres. The facilities for patient support were quite variable within each hospital group. The choice of mastectomy or conservative surgery was similar for all hospital groups. Breast reconstruction was rare in all hospital groups, and it seems unlikely that substantial numbers of patients refused reconstruction, as up to half of patients offered immediate breast reconstruction are reported to take up this offer (Watson et al, 1995).

Practice and patterns of referral may have changed between 1992 and the present. The screening centres report dramatic increases in workload but, until population-based data are complete, these reports cannot be substantiated. There was no trend in referral between 1991 and 1992, about 40% being referred to high-volume centres in both years (Thames Cancer Registry data). This may be because the screening units were all well established by 1991 in this region. There was little evidence of trends in the use of adjuvant chemotherapy and adjuvant radiotherapy treatment between our 1990 survey (Chouillet et al, 1994) and 1992. However, there was a marked increase in axillary surgery from 46% in 1990 to 64% in 1992, and the NHS breast screening programme has been influential in improving pathological reporting of breast cancers, as stage is essential for evaluation of the screening programme. Facilities for patient support have improved greatly since 1989, when there were very few breast care nurses (Fallowfield et al, 1992). By 1994, there were specialist nurses covering all high-volume, medium-volume and teaching hospitals and some low-volume hospitals.

© Cancer Research Campaign 1997

The results for the three university hospitals were perhaps surprising. In most respects, they were consistent with their (medium or low) volume group. They were atypical in low use of tamoxifen, poor reporting of tumour size, better than average clearance of the axilla, some very long waiting times for treatment and high usage of investigations such as bone scans. Substantial savings could be made by eliminating inappropriate investigations. These results are for patients referred first to the university surgeon; tertiary referrals for oncology probably form the larger part of the university workload and were not audited in this study.

It is extremely important for the success and acceptability of this type of work that the local providers and surgeons feel involvement and ownership and that they receive regular feedback and opportunity for discussion. The registry's role as a neutral and independent organization outside the purchaser–provider framework was a key factor in obtaining the agreement of all providers to participate in the study. A prospective audit of all breast patients is now being implemented which will inform purchasers, as part of the process of reorganizing cancer services, and will assist clinicians in tightening protocols and improving care and outcome for patients.

### ACKNOWLEDGEMENTS

We would like to thank the R&D directorate of North Thames RHA for funding this study; all members of the Clinical Advisory Panel for their hard work on the local guidelines and comments on the feedback and draft papers; and the surgeons in all the provider units without whose help this study would have been impossible. We are grateful to Sabina Moritz at the CRC Trials Unit in London for assisting in checking trial accrual. We are also indebted to Dr Ana Maria Chouillet for her work in setting up the study; Dr Jean-Michel Lutz, Mr Dunyou Tang, Mr Stephen Darlington and Mr Jason Hiscox for advice on the analysis of the study; and in particular the Thames Cancer Registry's tumour registrars without whose help this study would have been impossible.

### REFERENCES

Armitage P and Berry G (1987) *Statistical Methods in Medical Research*, 2nd edn. Blackwell: Oxford

Basnett I, Gill M and Tobias JS (1992) Variations in breast cancer management between a teaching and a non-teaching district. *Eur J Cancer* **28A**: 1945–1950

Bell CMJ, Basnett I, Ma M, Campbell S, Pollock A and Taylor I (1996) The use of local guidelines and regionwide audit to improve the management of breast cancer (submitted)

British Association of Surgical Oncology (BASO) (1995) Guidelines for surgeons in the management of symptomatic breast disease in the United Kingdom. *Eur J Surg Oncol* **21** (suppl. A): 1–13

Cancer Guidance sub-group of the Clinical Outcomes Group (1996) *Guidance for Purchasers – Improving outcomes in Breast Cancer*, report. Department of Health: London

Chouillet AM, Bell CMJ and Hiscox JG (1994) Management of breast cancer in Southeast England. *BMJ* **308**: 168–171

Davies HO and Crombie IK (1995) Assessing the quality of care. *BMJ* **311**: 766–766

Early Breast Cancer Trialist's Collaborative Group (1992) Systemic treatment of early breast cancer by hormonal, cytotoxic or immune therapy. *Lancet* **339**: 1–15

Expert Advisory Group on Cancer to the Chief Medical Officer of England and Wales (1995) *Consultative Document: A Policy Framework for Commissioning Cancer Services*. Department of Health: London

Fallowfield L and Roberts R (1992) Cancer counselling in the United Kingdom. *Psychol Health* **6**: 107–117

Gillis CR and Hole D (1996) Survival outcome of care by specialist surgeons in breast cancer: a study of 3786 patients in the west of Scotland. *BMJ* **312**: 145–148

Greco PJ and Eisenberg JM (1993) Changing physicians' practices. *N Engl J Med* **329**: 1271–1274

Grimshaw JM and Russell IT (1993) Effect of clinical guidelines on medical practice: a systematic review of rigorous evaluations. *Lancet* **342**: 1317–1322

Haines A and Jones R (1994) Implementing the findings of research. *BMJ* **304**: 1488–1492

Health Committee (1995) *Breast Cancer Services*. 3rd report, HMSO: London

HMSO (1989) *Working Paper 6. Medical Audit*. HMSO: London

International Agency for Research on Cancer (1995) *Survival of Cancer Patients in Europe. The EUROCARE Study*. IARC: Lyon

Institute of Physics & Engineering in Medicine (1993) *The Commissioning and Routine Testing of Mammography X-ray Systems*. IPSH: York

Joint Council for Clinical Oncology (1991) *Cancer Care and Treatment Services: Advice for Purchasers and Providers*. Royal College of Physicians and Royal College of Radiology: London

Joint Council for Clinical Oncology (1993) Reducing delays in cancer treatments. Royal College of Physicians and Royal College of Radiology: London

Joint Council for Clinical Oncology (1994) Improving quality in cancer care: quality control in cancer chemotherapy. Royal College of Physicians and Royal College of Radiology: London

King's Fund (1986) Consensus development conference: treatment of primary breast cancer. *BMJ* **293**: 946–947

NHSBSP (1989*a*) *QA Guidelines for Mammography (Pritchard Report)*. Royal College of Physicians and Royal College of Radiology: London

NHSBSP (1989*b*) *Pathology Reporting in Breast Cancer Screening*. Royal College of Physicians and Royal College of Radiology: London

NHSBSP (1993) *Guidelines for Nurses in Breast Cancer Screening*. Royal College of Physicians and Royal College of Radiology: London

Report of a Working Party of the British Breast Group (1994) *Provision of Breast Services in the UK: the Advantages of Specialist Breast Units*.

Richards MA, Wolfe CDA, Tilling K, Barton J, Bourne HM and Gregory WM (1996) Variations in the management and survival of women under 50 years with breast cancer in the South East Thames region. *Br J Cancer* **73**: 751–757

Sainsbury R, Haward B, Rider L and Johnston C (1995*a*) Influence of clinician workload and patterns of treatment on survival from breast cancer. *Lancet* **345**: 1265–1270

Sainsbury JRC, Rider L, Smith A and McAdam WFA (1995*b*) Does it matter where you live? Treatment variation for breast cancer in Yorkshire. *Br J Cancer* **71**: 1275–1278

Thames Cancer Registry (1994) Cancer incidence, prevalence and survival in residents of the district health authorities in South East England.

Watson JD, Sainsbury JRC and Dixon JM (1995) Breast reconstruction after surgery. *BMJ* **310**: 117–121

© Cancer Research Campaign 1997